# Dispersion Theory of Surface Plasmon Polaritons on Bilayer Graphene Metasurfaces

**DOI:** 10.3390/nano12111804

**Published:** 2022-05-25

**Authors:** Yong-Qiang Liu, Zhongru Ren, Hongcheng Yin, Jinhai Sun, Liangsheng Li

**Affiliations:** 1 Science and Technology on Electromagnetic Scattering Laboratory, Beijing 100854, China; yinhc207@126.com (H.Y.); jinhaisun@126.com (J.S.); liliangshengbititp@163.com (L.L.); 2 College of Information Engineering, Communication University of China, Beijing 100024, China; renzr0419@163.com

**Keywords:** surface plasmon polaritons, graphene metasurfaces, double layer, dispersion theory, modal characteristic, Terahertz applications

## Abstract

Surface plasmon polaritons (SPPs) on the graphene metasurfaces (GSPs) are crucial to develop a series of novel functional devices that can merge the well-established plasmonics and novel nanomaterials. Dispersion theory on GSPs is an important aspect, which can provide a basic understanding of propagating waves and further guidance for potential applications based on graphene metamaterials. In this paper, the dispersion theory and its modal characteristics of GSPs on double-layer graphene metasurfaces consisting of the same upper and lower graphene micro-ribbon arrays deposited on the dielectric medium are presented. In order to obtain its dispersion expressions of GSP mode on the structure, an analytical approach is provided by directly solving the Maxwell’s equations in each region and then applying periodical conductivity boundary onto the double interfaces. The obtained dispersion expressions show that GSPs split into two newly symmetric and antisymmetric modes compared to that on the single graphene metasurface. Further, the resultant dispersion relation and its propagating properties as a function of some important physical parameters, such as spacer, ribbon width, and substrate, are treated and investigated in the Terahertz band, signifying great potentials in constructing various novel graphene-based plasmonic devices, such as deeply sub-wavelength waveguides, lenses, sensors, emitters, etc.

## 1. Introduction

As one of the most important research fields in modern optics and nanophotonics, plasmonics has shown its strong vitality and lasting impact by taking advantage of the coupling between light and free carriers on the conducting and/or semi-conducting films [1,2,3,4]. The strong localization of surface plasmon polaritons (SPPs) beyond the diffraction limit is fundamental to develop a series of photonic, plasmonic, and optoelectronic devices [5,6]. However, conventional plasmonic structures are mostly based on the noble metals/dielectrics, and its obvious drawbacks, such as significant dissipative losses, limited mode response, and poor tunable capabilities, make it unsuitable for further high-performance plasmonic applications. Over the last decade, much more attention and effort has been devoted to the search for novel materials and structures which can sustain SPPs’ mode beyond conventional ones. Among these great progress and achievements [1,2,3,4,5,6,7,8], graphene stands out from various materials owing to its several advantages.

Graphene is a novel nanomaterial which is composed of an ultra-thin two-dimensional (2D) carbon atoms sheet within a honeycomb lattice. Its unique atomic arrangement results in a linearly carrier dispersion and ultra-high carrier mobility at room temperature [7,8]. These excellent properties of graphene make it very attractive to sustain and propagate SPPs’ mode, especially in the Terahertz (THz) band where its resonant plasma frequency locates exactly in this spectrum [9,10,11,12,13,14,15,16,17,18,19,20,21]. In these studies on the graphene SPPs (GSPs) and their applications, graphene is usually viewed as an infinite surface, thus the open space boundary condition on 2D sheet is commonly applied. Therefore, its fundamental dispersion theories are well-established and are commonly used for various applications, such as graphene sheet in the single- [9,10,11,12,13,14,15] or double-layer structures [16,17,18,19,20,21]. However, the uniform graphene sheet cannot meet increasing demand on the versatile material parameter change and the unprecedented electromagnetic control. Therefore, graphene metamaterials or metasurfaces with specific shapes are largely adopted and investigated in recent years [22,23,24,25,26,27,28,29,30,31,32,33,34,35,36,37,38,39,40,41,42,43]. Many studies show that graphene metasurfaces demonstrate more superior properties compared to those on the uniform sheet, such as enhanced transmission [22,23,24,25,26,27,28], more versatile wave control [29,30,31,32,33,34,35,36], the tighter field confinement [37,38,39,40,41,42], etc. A plethora of periodical graphene metasurfaces are proposed, such as graphene patches or ribbons [22,23,24,25,26,27], graphene rings, elliptical or circles [28,29,30,31,32], hollow-carved “H” shapes [33], sinusoidal shaped structure [35], and so on. Further, these graphene metasurfaces with periodical arrays are also created for some functional devices, such as waveguides [22,24,25], cloaks [26], sensors [29,37,40], absorbers [33,34,35], metalens [36], polarization splitter and converters [32,33], enhanced THz radiations sources [43], and many others.

Despite this progress on the graphene metasurfaces with various types of topological structures [22,23,24,25,26,27,28,29,30,31,32,33,34,35,36,37,38,39,40,41,42,43], it should be noted that most of these studies are based on numerical simulations, which heavily rely on some software studios. Nevertheless, numerical investigations based on Maxwell’s equations usually ignore the physical phenomena of SPPs behind the designed devices and sometimes are troublesome for some complicated metasurfaces. Thus, a fully analytic or quasi-analytic method of GSPs mode on the graphene metasurface is convenient and can help the detailed understanding of these light–matter interactions with various structural and material parameters [22,23,24,25,26,27,28,29,30,31,32,33,34,35,36,37,38,39,40,41,42,43]. Unfortunately, very limited works are conducted to this goal and the mode analysis on the graphene metasurface is usually based on the transfer matrix method or a simplified scaling law [22,23,24]. Some rigorous analysis on the GSP dispersion theory and the concise dispersion expression is revealed, provided and reported by some of the recent literature [44,45,46,47,48]. In Ref. [44], the authors provide accurate plasmon modes analysis of periodical nanoribbon arrays by using a time-dependent density theory. Further, they also deal with two different graphene nanoribbon edges (i.e., Zigzag and Armchair), and different dispersion modes are also found. In Ref. [47], the authors provide a unified dispersion theory of periodical ribbon arrays by merging different ribbon edges via periodical conductivity boundary into Maxwell’s equations. Thus, a simple and concise dispersion expression is revealed and is also applied to analyze various GSP modes with different parameters. The dispersion expression and mode distribution are simple for the single graphene metasurface [44,47]. Very recently, some functional plasmonic devices have also been designed on double-layer graphene metasurfaces in [49,50,51,52,53,54,55,56]. Compared to single-layer graphene metasurface, a double-layer structure can provide more degrees of freedom to control GSPs and, thus, may be more advantageous than a single-layer structure [52,53,54]. However, its fundamental dispersion theory and the detailed GSPs’ mode distributions with various parameters are not reported so far because of its complicated periodical boundary conditions.

In this paper, the dispersion theory and its mode distributions on double-layer graphene metasurfaces with various physical parameters are presented, studied, and analyzed in THz band. The general dispersion theory is firstly provided by treating different field expressions in each region, along with the periodical boundary condition on double-layer graphene metasurfaces. Following these fully analytical expressions with different dispersion characteristics, its GSP dispersion diagrams and the mode distributions are studied with different physical parameters. Its potential applications based on double-layer graphene metasurfaces are also envisioned and discussed.

## 2. Dispersion Theory on Double-Layer Graphene Metasurfaces

### 2.1. Model and Theory

**Figure 1:**
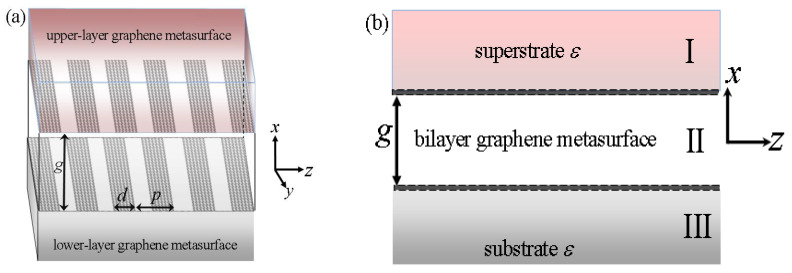
(**a**) The schematic diagrams of the bi-layer grapehene metasurfaces which are deposited on the same superstrate and substrate medium ε. The upper- and lower-layer graphene metasurface has the same width *d* and period *p*. The bi-layer graphene metasurface is separated by an air gap with depth of *g*. (**b**) The cross-section views of the bi-layer graphene metasurfaces on x–z plane. The whole structure is divided by three different regions, as labeled by I, II, and III, for the studies on its dispersion theory.

Distinct to previously double-layer graphene sheet with homogeneous boundary on the interface [16,17,18,19,20,21], GSP modes on the periodical graphene metasurfaces are more complicated and the derivation processes are also very challenging by taking into account the inhomogeneity of surface boundary conditions. According to waveguide mode theory [57,58], the electromagnetic fields in the periodical structure should include Bloch’s waves and the dispersion mode also repeats itself in the dispersion diagrams. We here take the most simple graphene metasurface of periodical rectangular ribbon arrays on double-layer structure into account on the 2D space. The studies can also be extended to other more complicated structures for 3D applications. In addition, an evanescently transverse magnetic polarization (TM)-guided mode is considered for simplicity and the theory can also be applied to transverse electric polarization (TE) if different graphene conductivity is used [20,21]. The considered theoretical model of the bi-layer graphene metasurfaces is plotted in Figure 1a. The upper- and lower-layer graphene metasurface of periodical ribbon arrays deposited on the identical dielectric medium *ε* has the same structural parameters of width *d* and period *p*. The bi-layer metasurfaces align with each other in both longitudinal and vertical directions and are separated by an air gap depth, as denoted by *g*. Its cross-section views of double-layer structure are schematically plotted in Figure 1b on the x–z plane, and the edge effect along y axis can be ignored [16,17,18,19,20,21] because of its intrinsic 2D properties of GSPs.

The key to obtaining its dispersion expressions on double-layer graphene metasurfaces is to apply the periodical surface conductivity to the different field expressions in each region, as indicated by I, II, and III in Figure 1b. The zero point of the x axis locates at the center of the air gap. The zero point of the z axis is defined at the left edge of one graphene ribbon. The surface conductivity formulism of graphene along the z axis is, thus, given as below:

(1) $$\sigma_{g}=\left\{ \begin{array}{l} \sigma (\omega ),mp<z<mp+d\\ 0,mp+d<z<mp+p \end{array}\right.$$

where *σ*(*ω*) is the frequency-dependent surface conductivity distribution of complete graphene sheet and m is the arbitrary integer. The surface conductivity of graphene has been widely applied [16,17,18,19,20,21,22,23,24,25,26,27,28,29,30,31,32,33,34,35,36,37,38,39,40,41,42,43], which includes the intraband and interband conductivity according to the well-known Kubo formula within random-phase approximation, i.e., *σ*(*ω*) = *σ^intra^* + *σ^inter^*. For intraband conductivity, it is given as:

(2) $$\sigma^{intra}=\frac{e^{2}}{\pi \hbar^{2}}\frac{2k_{B}T}{\tau^{-1}-j\omega }In[2\cosh(\frac{\mu_{c}}{2k_{B}T})]\;$$

For interband conductivity, it can be approximated by the following under |*μ_c_*| > > |*k*_B_T|:

(3) $$\sigma^{inter}\approx -\frac{je^{2}}{4\pi \hbar }In\frac{2\left|\mu_{c}\right|-(\omega -j\tau^{-1})\hbar }{2\left|\mu_{c}\right|+(\omega -j\tau^{-1})\hbar }\;$$

The above surface conductivity of graphene is a function of radian frequency ω = 2π*f*, chemical potential *μ*_c_ (or Fermi level), electron relaxation time *τ* (*τ*^−^^1^ is scatter rate), and temperature T. Some other constant parameters are electron charge *e*, reduced Planck’s value *ħ*, and Boltzmann constant *k*_B_. *j* is an imaginary unit. The intraband and interband conductivity can be simultaneously applied to graphene metasurface according to different operation frequency spectra. For some studies in the low frequency band, such as THz [22,23,24,25,26,27,28,29,30,31,32,33,34,35,36,37,38,39,40,41,42,43], the intraband conductivity can represent its surface conductivity of graphene because of its dominant intraband transition process.

### 2.2. Field Expression Derivations in Each Region

In order to obtain its dispersion equations on double-layer graphene metasurfaces in Figure 1, the field expressions should be solved first in each different region, as indicated by label I, II, and III in Figure 1b. For the electromagnetic fields in region I and III, they should have the similar forms because of the totally defined symmetric conditions. The electric fields can be obtained by solving Maxwell’s equations based on the above assumptions when the dielectric medium possesses an infinitely thick depth. In region I (*x* > *g*/2), its electric field along the z direction and magnetic fields along the y direction are as follows:

(4) $$E^{I}{}_{z}=\sum_{n=-\infty }^{\infty }A_{n}e^{-\kappa (x-\frac{g}{2})}e^{{}^{-j\beta_{n}z}}$$

(5) $$H^{I}{}_{y}=\sum_{n=-\infty }^{\infty }A_{n}\frac{j\omega \varepsilon }{\kappa }e^{-\kappa (x-\frac{g}{2})}e^{{}^{-j\beta_{n}z}}$$

Moreover, the field expressions in region III (*x* < −*g*/2) can also be obtained similarly, as below:

(6) $$E^{\mathrm{III}}{}_{z}=\sum_{n=-\infty }^{\infty }B_{n}e^{\kappa (x+\frac{g}{2})}e^{{}^{-j\beta_{n}z}}$$

(7) $$H^{\mathrm{III}}{}_{y}=\sum_{n=-\infty }^{\infty }-B_{n}\frac{j\omega \varepsilon }{\kappa }e^{\kappa (x+\frac{g}{2})}e^{{}^{-j\beta_{n}z}}$$

The fields inside the upper and lower dielectric medium are periodical by the Block’s harmonic waves along propagation direction z. *A_n_* and *B_n_* are the unknown index and *β_n_* = *β*_0_ + 2nπ/*p* (*n* = 0, ±1, ±2, ±3…) denotes its propagation constant of the considered harmonic mode of *n* along the propagation direction. *κ* is the decay coefficient in the perpendicular direction, as calculated by *κ^2^* = *β_n_*^2^ − *εk*^2^, *k* = *ω*/c is the wave vector in free space, and c is light velocity.

In the air gap region II, which is between upper- and lower-layer graphene metasurface (−*g*/2 < x < *g*/2), its electromagnetic fields obey the following expressions:

(8) $$E^{\mathrm{II}}{}_{z}=\sum_{n=-\infty }^{\infty }[C_{n}\sinh(Kx)+D_{n}\cosh(Kx)]e^{{}^{-j\beta_{n}z}}$$

(9) $$H^{\mathrm{II}}{}_{y}=\sum_{n=-\infty }^{\infty }-\frac{j\omega \varepsilon_{0}}{\kappa }[C_{n}\cosh(Kx)+D_{n}\sinh(Kx)]e^{{}^{-j\beta_{n}z}}$$

where *C_n_* and *D_n_* are the unknown index and *ε*_0_ is air permittivity. *κ* is the decay coefficient in the perpendicular direction. *κ*^2^ = *β_n_*^2^ − *k*^2^. Other symbols are the same as those in Equations (4)–(7). sinh and cosh are hyperbolic sine and cosine functions, respectively. The similar derivation processes and obtained field expressions are also successfully applied to some other double-layer SPP structures, such as corrugated metallic waveguide [59,60,61,62]. In addition, the concise dispersion expressions are also revealed for symmetric and nonsymmetric conditions in [59,60] and [61,62,63], respectively.

### 2.3. Dispersion Expressions of GSP Mode

Based on the above electromagnetic fields Equations (4)–(9), in region I, II, and III, the periodical surface conductivity and tangential electric field boundary conditions on the upper interface of *x* = *g*/2 and the lower interface of *x* = −*g*/2 should be mandatorily satisfied. For the upper graphene metasurface between region I and II (*x* = *g*/2 plane), the following boundary conditions should be obeyed:

(10) $$E^{I}{}_{z}|{}_{x=g/2}=E^{\mathrm{II}}{}_{z}|{}_{x=g/2}$$

(11) $$(H^{I}{}_{y}-H^{\mathrm{II}}{}_{y})|{}_{x=g/2}=\sigma_{g}E^{I}{}_{z}|{}_{x=g/2}$$

Moreover, for the lower graphene metasurface between region III and II (*x* = −*g*/2 plane), there should be following expressions:

(12) $$E^{\mathrm{III}}{}_{z}|{}_{x=-g/2}=E^{\mathrm{II}}{}_{z}|{}_{x=-g/2}$$

(13) $$(H^{\mathrm{II}}{}_{y}-H^{\mathrm{III}}{}_{y})|{}_{x=-g/2}=\sigma_{g}E^{\mathrm{III}}{}_{z}|{}_{x=-g/2}$$

Applying the electric field of Equations (4), (6) and (8) in region I, III, and II to the boundary conditions of (10) and (12), the following equations are obtained:

(14) $$\sum_{n=-\infty }^{\infty }[C_{n}\sinh(K\frac{g}{2})+D_{n}\cosh(K\frac{g}{2})]e^{{}^{-j\beta_{n}z}}=\sum_{n=-\infty }^{\infty }A_{n}e^{{}^{-j\beta_{n}z}}$$

(15) $$\sum_{n=-\infty }^{\infty }[C_{n}\sinh(-K\frac{g}{2})+D_{n}\cosh(-K\frac{g}{2})]e^{{}^{-j\beta_{n}z}}=\sum_{n=-\infty }^{\infty }B_{n}e^{{}^{-j\beta_{n}z}}$$

Furthermore, taking the magnetic field of Equations (5), (7) and (9) in region I, III, and II to the boundary conditions of (11) and (13), we have the following equations:

(16) $$\begin{array}{l} \sum_{n=-\infty }^{\infty }A_{n}\frac{j\omega \varepsilon }{\kappa }e^{{}^{-j\beta_{n}z}}+\sum_{n=-\infty }^{\infty }\frac{j\omega \varepsilon_{0}}{\kappa }[C_{n}\cosh(K\frac{g}{2})+D_{n}\sinh(K\frac{g}{2})]e^{{}^{-j\beta_{n}z}}\\ =\sigma_{g}\sum_{n=-\infty }^{\infty }A_{n}e^{{}^{-j\beta_{n}z}} \end{array}$$

(17) $$\begin{array}{l} \sum_{n=-\infty }^{\infty }-\frac{j\omega \varepsilon_{0}}{\kappa }[C_{n}\cosh(-K\frac{g}{2})+D_{n}\sinh(-K\frac{g}{2})]e^{{}^{-j\beta_{n}z}}+\sum_{n=-\infty }^{\infty }B_{n}\frac{j\omega \varepsilon }{\kappa }e^{{}^{-j\beta_{n}z}}\\ =\sigma_{g}\sum_{n=-\infty }^{\infty }B_{n}e^{{}^{-j\beta_{n}z}} \end{array}$$

These four Equations (14)–(17) are the foundations of final GSP dispersion expression results. Obviously, the challenges are eliminating these four unknown indexes of *A_n_*, *B_n_*, *C_n_*, and *D_n_* in Equations (14)–(17). The Equations (14) and (15) can be readily transferred into the following simple Equations (18) and (19), respectively. By integrating the periodical surface conductivity in Equations (16) and (17) in one period according to expression (1), the following Equations (20) and (21) concise equations also arise after some mathematic treatments:

(18) $$C\sinh(K\frac{g}{2})+D\cosh(K\frac{g}{2})=A$$

(19) $$C\sinh(-K\frac{g}{2})+D\cosh(-K\frac{g}{2})=B$$

(20) $$\begin{array}{l} A_{n}\frac{j\omega \varepsilon }{\kappa }+\frac{j\omega \varepsilon_{0}}{\kappa }[C_{n}\cosh(K\frac{g}{2})+D_{n}\sinh(K\frac{g}{2})]\\ =\sigma (\omega )\sum_{n=-\infty }^{\infty }A_{n}\frac{\sin(\frac{\beta_{n}}{2}d)}{\sin(\frac{\beta_{n}}{2}p)} \end{array}$$

(21) $$\begin{array}{l} -\frac{j\omega \varepsilon_{0}}{\kappa }[C_{n}\cosh(-K\frac{g}{2})+D_{n}\sinh(-K\frac{g}{2})]+B_{n}\frac{j\omega \varepsilon }{\kappa }\\ =\sigma (\omega )\sum_{n=-\infty }^{\infty }B_{n}\frac{\sin(\frac{\beta_{n}}{2}d)}{\sin(\frac{\beta_{n}}{2}p)} \end{array}$$

It should be noted that the periodical surface conductivity of graphene in Equations (16) and (17) with σ_g_ now become the well-known infinite surface conductivity of *σ(ω*) in Equations (20) and (21) with the help of our special calculations. Finally, the expressions of *A_n_* and *B_n_* in Equations (18) and (19) can be directly applied to Equations (20) and (21). Thus, the four unknown indexes are reduced to only *C_n_* and *D_n_* within two equations. If we solve these two equations by eliminating the two unknown *C_n_* and *D_n_*, the final GSP dispersion expression can be obtained directly in the proposed double-layer graphene metasurfaces system. For symmetric GSP mode, we have the following expression:

(22) $$\sum_{n=-\infty }^{\infty }\frac{j\omega \varepsilon }{\kappa }\tanh(K\frac{g}{2})+\sum_{n=-\infty }^{\infty }\frac{j\omega \varepsilon_{0}}{K}=\sum_{n=-\infty }^{\infty }\tanh(K\frac{g}{2})\sigma \frac{\sin(\frac{\beta_{n}}{2}d)}{\sin(\frac{\beta_{n}}{2}p)}$$

For antisymmetric GSP mode, its analytical expression is:

(23) $$\sum_{n=-\infty }^{\infty }\frac{j\omega \varepsilon }{\kappa }\coth(K\frac{g}{2})+\sum_{n=-\infty }^{\infty }\frac{j\omega \varepsilon_{0}}{K}=\sum_{n=-\infty }^{\infty }\coth(K\frac{g}{2})\sigma \frac{\sin(\frac{\beta_{n}}{2}d)}{\sin(\frac{\beta_{n}}{2}p)}$$

The symbols in the analytical dispersion expressions are the same as those in the above solved field expressions. The obtained concise dispersion equations with frequency-wave vector relation include some structural and material parameters and, thus, provide a simple yet useful tool to analyze the basic propagation properties of GSP mode on double-layer graphene metasurfaces in Figure 1. Further, they can also be readily used to develop some novel functional devices [49,50,51,52,53,54,55,56] if the specific dispersion diagrams are calculated.

## 3. Results and Discussions

### 3.1. Validation of the Proposed Dispersion Theory on the Structure

Although the dispersion theory and its expressions on double-layer graphene structure without periodical patterns have long existed in the literature, such as [16,17,18,19,20,21], we here address the dispersion theory of double-layer graphene metasurfaces, thus its scope is largely extended and becomes more general. GSPs’ mode on the periodical structure is more complicated, which includes Block’s harmonic waves, compared to that on the uniform interface. If we assume the proposed double-layer structure degraded into the non-periodical one with condition *d* = *p*, the results of (22) and (23) become the simple one, which has been discussed in [16,17,18]. Although the similar method and calculations are used, its derivation process is more challenging and tedious compared to previous ones on the uniform structure [16,17,18,19,20,21]. Furthermore, GSPs’ mode splits into two new symmetric and antisymmetric modes compared to that on the single graphene metasurface [44,47]; thus, the light–matter interaction on a double-layer structure also becomes complex. Actually, if the gap size between our proposed double-layer structures becomes large enough with *g* as infinite in Equations (22) and (23), GSP mode on the upper- and lower-layer one will decouple with each other; thus, the two different modes become the unified single one with the same dispersion expression as shown in [47]. Further, the proposed dispersion theory on double-layer graphene metasurfaces can be further verified by some numerical simulations, such as FDTD in [43] and other software studios in [59,60,61,62,63], where the similar derivation processes are applicable.

### 3.2. Dispersion Diagrams of GSP Mode on Double-Layer Structure with Different Parameters

**Figure 2:**
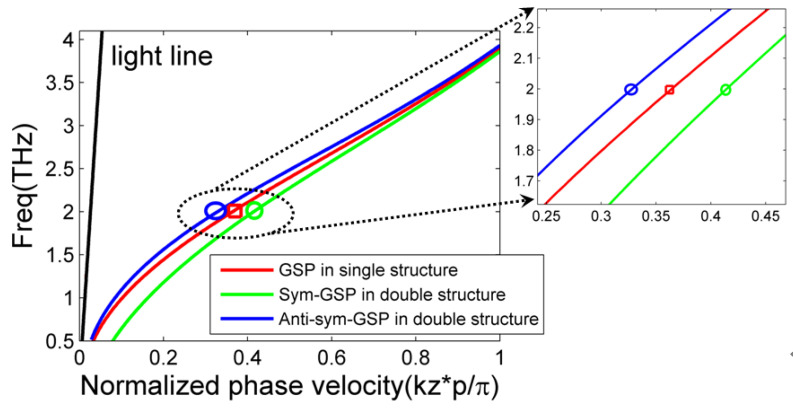
Dispersion diagrams of the GSP mode on the single- (**red line**) and double-layer (**blue and green line**) graphene metasurfaces. For graphene metasurface, its width and period are 0.5 and 1 μm, respectively. The gap size of bi-layer structure is *g* = 1 μm. Others are given in the text.

As a specific illustration of the GSP mode on double-layer graphene metasurfaces, the transcendental dispersion equations are solved and its curve lines with symmetric and antisymmetric mode are plotted in Figure 2 with green and blue lines, respectively. The graphene metasurface structural parameters in Figure 1 are: *d* = 0.5 μm, *p* = 1 μm, and g = 1 μm. Moreover, the graphene material parameters are also set as constant as: μ_c_ = 0.1 eV, ***τ*** = 0.5 ps, and T = 300 K. The upper and lower dielectric is silica with permittivity of *ε* = 3.92 in THz band [47,49,50,51,52]. It can be noted that symmetric GSP mode occupies its location in the lower band, while antisymmetric mode in the upper band. The phase velocity of symmetric mode is larger than antisymmetric mode at the given operation frequency. In addition, symmetric and antisymmetric GSP modes gradually converge into the same asymptotical frequency of around 4 THz as the phase velocity increases. As a comparison with GSP mode on the single graphene metasurface, its dispersion curve is also plotted as a red line and its parameters are the same with bi-layer metasurfaces [47]. The oblique black line is light dispersion in the dielectric. The obtained simulated asymptotical frequency is around 7.5 THz, which is larger than the theoretical one. In the simulations by CST Microwave studio suite (version is 2018), the periodical condition along the z direction is applied and the parameters are the same with the theoretical model (dielectric thickness is 50 μm).

**Figure 3:**
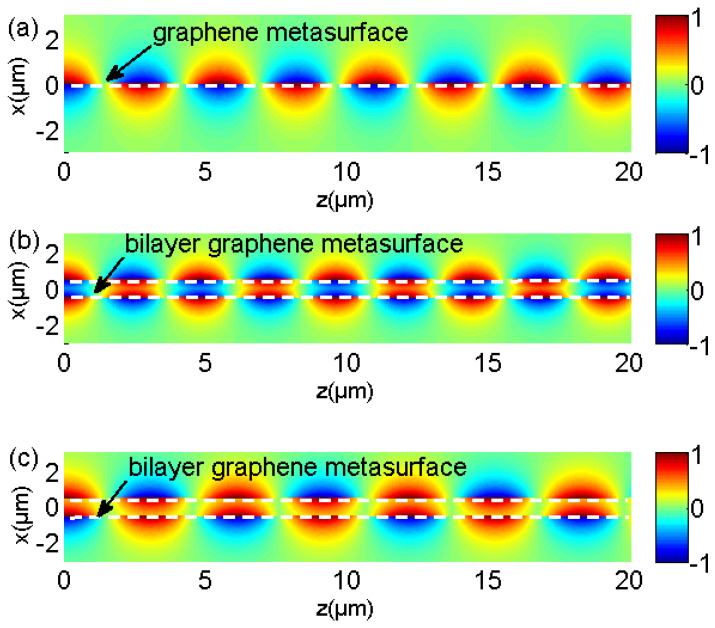
The electric field patterns of GSP mode on the (**a**) single- and (**b**,**c**) double-layer graphene metasurfaces at 2 THz as indicated in the inset of Figure 2. The white line denotes the location of each graphene metasurface.

Compared to GSP mode on the single graphene metasurface, GSP mode on double-layer structure splits into two newly symmetric and antisymmetric modes, which locate on the different regions of dispersion diagrams. Further, its phase velocity difference becomes larger in the middle of dispersion lines, as illustrated in the inset of Figure 2. Its detailed modal patterns at 2 THz with single- and double-layer graphene metasurfaces are plotted specifically in Figure 3a–c, respectively. The structural and material parameters are the same as those of Figure 2. It can be clearly noted that symmetric and antisymmetric GSPs’ modal profile occurs for symmetric and antisymmetric GSP modes, respectively. The normalized field patterns are calculated from previous analytical electric field expressions.

**Figure 4:**
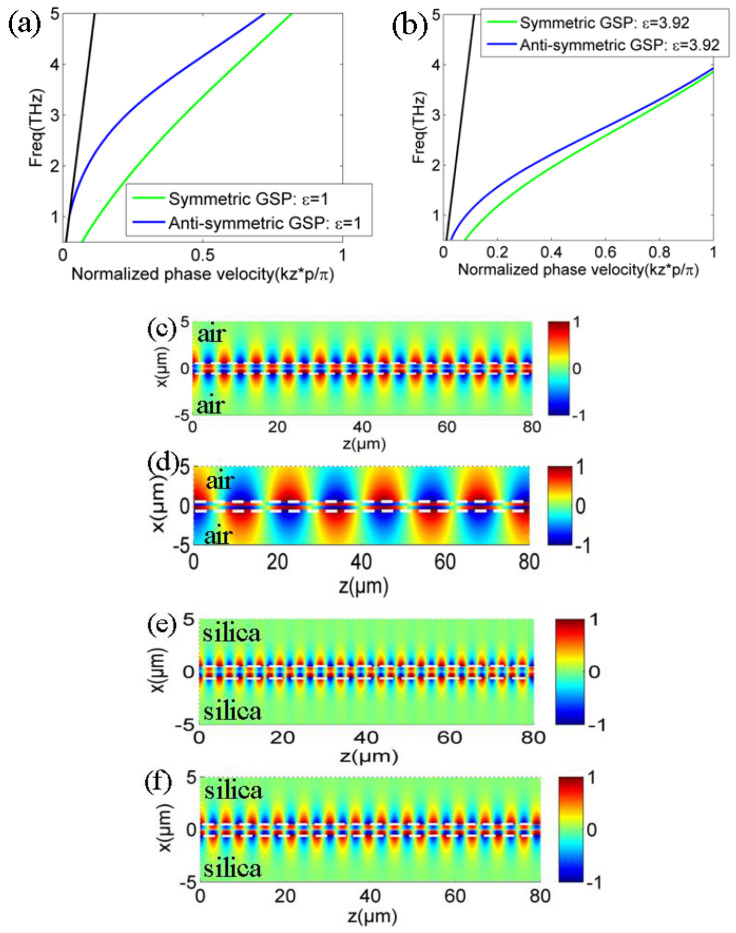
(**a**,**b**) are the GSP dispersion lines on double-layer graphene metasurfaces with dielectric ε = 1 (air) and ε = 3.92 (silica) conditions in Figure 1. (**c**,**d**) are the symmetric and antisymmetric GSP mode patterns with ε = 1 (air) condition, respectively. (**e**,**f**) are the corresponding GSP modes with ε = 3.92 (silica), respectively. Operation frequency is at 2 THz and other parameters are given in text.

**Figure 5:**
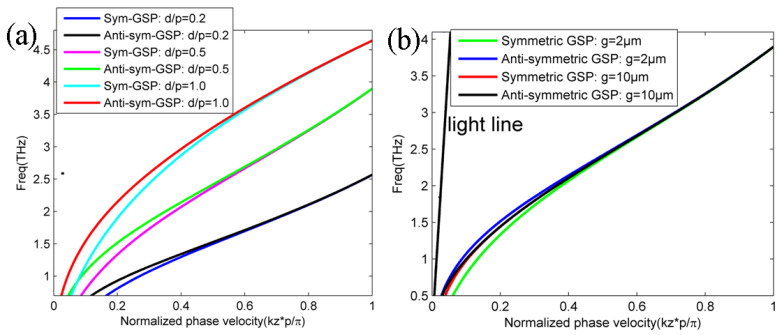
Symmetric and antisymmetric GSP dispersion diagrams with different (**a**) graphene ribbon width *d* and (**b**) gap size *g* between upper and lower graphene metasurfaces in Figure 1. Other parameters are the same as those in Figure 2.

GSP dispersion diagrams and its propagation characteristic on double-layer graphene metasurfaces can be obtained and analyzed with arbitrary structural parameters based on the obtained analytic dispersion Equation of (22) and (23). In addition, the mode analysis and dispersion engineering lay the foundations for further applications based on the gradient index distributions, which rely on the structural parameter changes of double-layer graphene metasurface, such as graphene lenses, couplers, emitters, absorbers, and sensors. Here, we consider different dispersion modes with various dielectric mediums, spacer, and graphene ribbon widths on the structure. Figure 4a,b plot the symmetric and antisymmetric GSP modes on double-layer graphene metasurfaces with different dielectric medium of *ε* = 1.0 (air) and *ε* = 3.92 (silica) in Figure 1, respectively. Similar to the dispersion diagrams and modal characteristic of SPP mode on the single-layer graphene metasurface in [47], the phase velocity of both symmetric and antisymmetric GSP modes become larger with the increased dielectric permittivity. The increased phase velocity and the enlarged modal departure from light line are very intriguing to create some ultra-compact and integrated devices, such as low-profile waveguide and emitters where a deeply sub-wavelength profile is highly valuable. In addition, the coupling coefficient of its absolute value between double-layer graphene metasurfaces becomes smaller with the increased filled dielectric permittivity as the phase difference of symmetric and antisymmetric SSP modes gradually vanishes [17,59]. In order to further demonstrate its different modal characteristics of GPS mode on double-layer graphene metasurfaces with different dielectric mediums, its normalized electric field patterns are also plotted in Figure 4c–d and Figure 4e–f with *ε* = 1.0 (air) and *ε* = 3.92 (silica), respectively. It is clarified that the field concentrations of symmetric and antisymmetric GSP modes with silica cladding are better than thosr of GSP mode with air cladding. The locations of upper and lower graphene metasurface are indicated by the dotted white lines. The superstrate and substrate medium are clearly marked in the structure. Finally, we examine the influence of structural parameters of graphene ribbon width *d*/*p* and the gap size *g* on the propagating GSP mode on double-layer structure. Figure 5a,b plot the dispersion lines of symmetric and antisymmetric GSP modes with different graphene ribbon width and gap size between bi-layer graphene metasurfaces according to our previously demonstrated dispersion theory in Equations (22) and (23), respectively. It can be noted that both symmetric and antisymmetric GSP dispersion line shift lower with the decreased graphene ribbon width from Figure 5a. For the calculation results in Figure 5b, symmetric GSP mode shifts lower for the decreased gap size, while the antisymmetric GSP mode shifts higher oppositely. The dispersion characteristic change in GSP mode is more sensitive by changing graphene ribbon width compared to the variation in gap size for the given structure. This also suggests that changing graphene ribbon width is more effective for creating some gradient-index metadevices where the full phase coverage is needed, such as high-efficiency couplers, meta-lenses, and enhanced emitters [49,50,51,52,53,54,55,56]. The analytic dispersion expressions are very useful to analyze its propagating characteristic and can also be directly used to design some novel graphene devices where its principle is to heavily rely on software simulations [49,50,51,52].

## 4. Conclusions

In this paper, the dispersion theory and its model properties of GSP mode on double-layer graphene metasurfaces are provided by solving the Maxwell’s equations combined with the periodical surface conductivity boundary conditions on the structure. Further, the analytical dispersion expression for symmetric and antisymmetric GSP modes is obtained and its detailed mode analysis with different structural dielectric and graphene ribbon parameters are presented in THz band. The dispersion theory on the bi-layer graphene metasurface provides a fast and powerful tool to understand and analyze its fundamental propagation characteristic of GSP mode on the complicated structured metasurfaces. The proposed dispersion theory on GSPs with double-layer graphene metasurfaces can be further verified by some numerical simulations and experimental measurements in the future. In addition, its presented dispersion characteristic is fundamental to design some novel THz devices on the bi-layer structure, such as couplers, meta-lenses, transmit-array, emitters, etc., in the future.

## Data Availability

Not applicable.
